# Unprecedented hypoxaemia caused by ventricular septal aneurysm protruding into left ventricular outflow tract in an adult with congenitally corrected transposition of the great arteries

**DOI:** 10.1093/ehjcr/ytae260

**Published:** 2024-05-21

**Authors:** Daiki Toyoshima, Yasuhide Mochizuki, Hideshi Tomita, Toshiro Shinke

**Affiliations:** Division of Cardiology, Department of Medicine, Showa University School of Medicine, 1-5-8 Hatanodai, Shinagawa-ku, Tokyo 142-8555, Japan; Division of Cardiology, Department of Medicine, Showa University School of Medicine, 1-5-8 Hatanodai, Shinagawa-ku, Tokyo 142-8555, Japan; Pediatric Cardiology and Adult Congenital Heart Disease Center, Showa University Hospital, 1-5-8 Hatanodai, Shinagawa-ku, Tokyo 142-8555, Japan; Division of Cardiology, Department of Medicine, Showa University School of Medicine, 1-5-8 Hatanodai, Shinagawa-ku, Tokyo 142-8555, Japan

The case involves a 48-year-old woman diagnosed with congenitally corrected transposition of the great arteries (ccTGA) complicated by left ventricular outflow tract (LVOT) obstruction, ventricular septal defect (VSD), and tricuspid regurgitation (TR) at age 3. The VSD closed spontaneously at age 24, without any surgical intervention to date. A detailed examination was conducted to investigate dyspnoea along with a decline in oxygen saturation below 90% in room air. Transthoracic echocardiography (TTE) revealed an eccentric mitral regurgitation (MR) in the right atrium (RA) and moderate systemic TR in the left atrium. The maximum pressure gradient at LVOT in TTE was 75 mmHg, and MR gradient measured between left ventricle and RA was 77 mmHg. However, the underlying cause of hypoxaemia remained unclear. Cardiac magnetic resonance imaging showed right to left shunt at the atrial level, which was considered a possible cause of hypoxaemia. Systemic right ventricular ejection fraction was preserved at 59%. Transoesophageal echocardiography (TEE) revealed a ventricular septal aneurysm (*Panel B*, yellow arrow) formed after the closure of a perimembranous VSD; it protruded from the right ventricle into the LVOT and caused LVOT stenosis and excessive systolic anterior motion of the mitral valve (*Panel B*, white arrow), resulting in eccentric MR jet (*Panels A–C*; see Supplementary material online, *Video S1*). Transoesophageal echocardiography further showed an advanced right-to-left shunt via an atrial septal defect (ASD) without the Valsalva manoeuver (*Panel D*; see Supplementary material online, *Video S2*). A schema summarizing data from TEE and right heart catheterization demonstrated that RA pressure was higher than PCWP in both atrial and ventricular systolic phases (*Panel E*). After trans-catheter closure of ASD, saturation increased to 97% in room air and her respiratory distress disappeared. An extremely rare condition of hypoxaemia was dynamically and three-dimensionally visualized by TEE in a patient with ccTGA.

**Figure:**
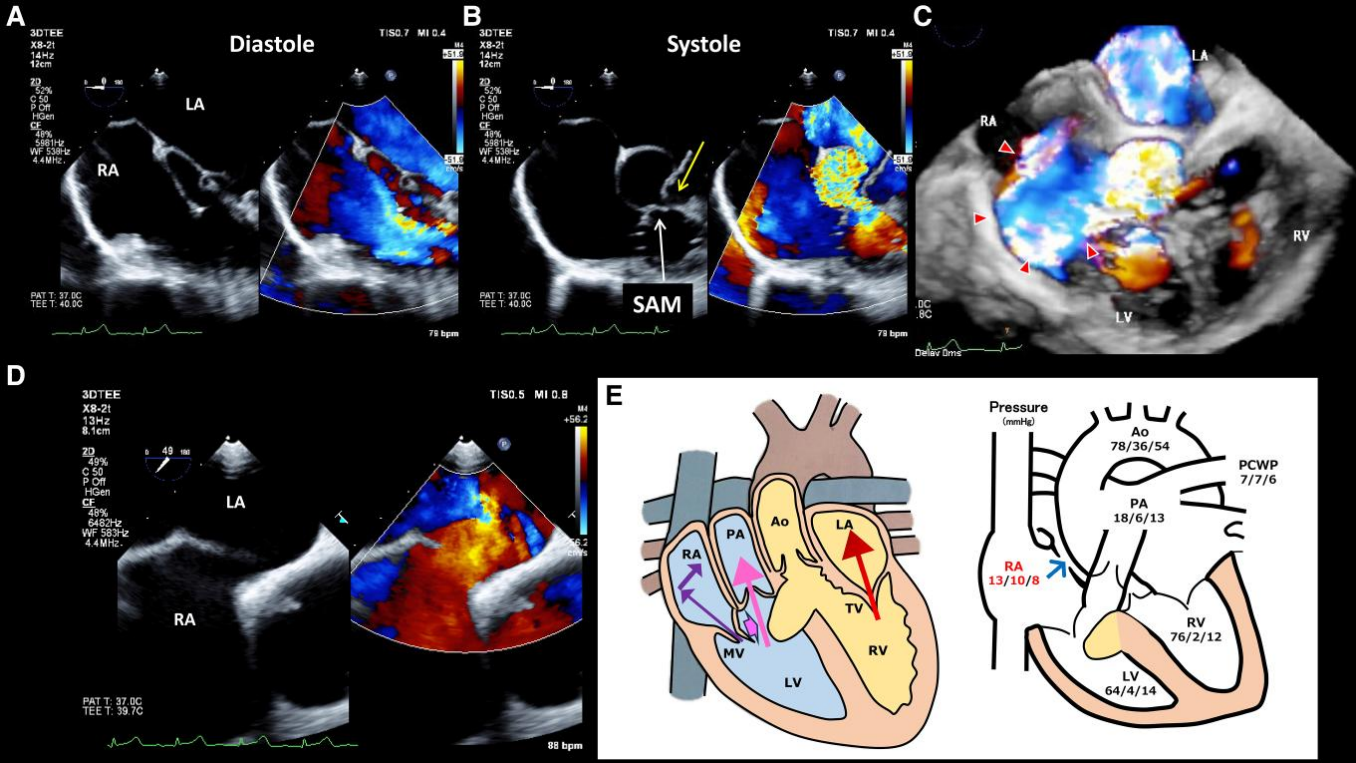


## Supplementary Material

ytae260_Supplementary_Data

## Data Availability

The data underlying this article will be shared on reasonable request to the corresponding author.

## Supplementary material

**Consent:** The written informed consent for publication was obtained from the patient.

**Funding:** None declared.

